# Highly Efficient DSSCs Sensitized Using NIR Responsive Bacteriopheophytine-a and Its Derivatives Extracted from Rhodobacter Sphaeroides Photobacteria

**DOI:** 10.3390/molecules29050931

**Published:** 2024-02-21

**Authors:** Abdulrahman I. Almansour, Raju Suresh Kumar, Khloud Ibrahim Al-Shemaimari, Natarajan Arumugam

**Affiliations:** Department of Chemistry, College of Science, King Saud University, P.O. Box 2455, Riyadh 11451, Saudi Arabia; almansor@ksu.edu.sa (A.I.A.); 442203282@student.ksu.edu.sa (K.I.A.-S.)

**Keywords:** DSSC, natural dye, Rhodobacter Sphaeroides, photosensitizer, TiO_2_

## Abstract

Employing naturally extracted dyes and their derivatives as photosensitizers towards the construction of dye-sensitized solar cells (DSSCs) has been recently emerging for establishing sustainable energy conversion devices. In this present work, Rhodobacter Sphaeroides Photobacteria (Rh. Sphaeroides) was used as a natural source from which Bacteriopheophytine-a (Bhcl) dye was extracted. Further, two cationic derivatives of Bhcl, viz., Guanidino-bacteriopheophorbide-a (Gua-Bhcl) and (2-aminoethyl)triphenylphosphono-bacteriopheophorbide-a (2AETPPh-Bhcl) were synthesized. The thus obtained Bhcl, Gua-Bhcl and 2AETPPh-Bhcl were characterized using liquid chromatography–mass spectrometry (LC–MS) and their photophysical properties were investigated using excitation and emission studies. All three near-infrared (NIR) responsive dyes were employed as natural sensitizers towards the construction of DSSC devices, using platinum as a photocathode, dye-sensitized P25-TiO_2_ as a photoanode and I^−^/I_3_^−^ as an electrolyte. DSSCs fabricated using all three dyes have shown reasonably good photovoltaic performance, among which 2AETPPh-Bhcl dye has shown a relatively higher power conversion efficiency (*η*) of 0.38% with a short circuit photocurrent density (*J*_SC_) of 1.03 mA cm^−2^. This could be attributed to the dye’s natural optimal light absorption in the visible and NIR region and uniform dispersion through the electrostatic interaction of the cationic derivatives on the TiO_2_ photoanode. Furthermore, the atomic force microscopy studies and electrochemical investigations using cyclic voltammetry, electrochemical impedance spectroscopy and Bode’s plot also supported the enhancement in performance attained with 2AETPPh-Bhcl dye.

## 1. Introduction

In recent years, low production costs and ecologically favorable operation have made dye-sensitized solar cells (DSSCs) an immensely attractive energy conversion device. The working of DSSC is comparable to the natural photosynthetic approach and, herein, the system is able to produce energy by transforming absorbed solar energy into its electrical form. The DSSC device is typically constructed using a dye sensitizer, a metal oxide-based semiconductor, an electrolyte comprising iodide/triiodide ions and a counter electrode [1,2,3]. The first DSSC patent was submitted in 1977 in the US, in which Prof. Michael Grätzel reported TiO_2_ with a polypyridyl complex of ruthenium as an anode. After several efforts, the efficiency of a small area cell has increased from 10% in 1990 to 11.9% in 2012, whereas with large-scale devices, it hardly reaches 5%. Hence, in order to enhance the efficiency, detailed research was carried out on rationally designing the individual components, which include photoanodes, counter electrodes, electrolytes and sensitizers [4,5]. Especially, sensitizers are essential for converting solar energy (photons) into electrical energy (electrons) in DSSC, and thus, numerous metal complexes, organometallics, organic dyes and derivatives were designed and employed as sensitizers. However, ruthenium-based synthetic dyes have exhibited superior performance as sensitizers, and especially, the DSSCs sensitized using Ru-based N719 have demonstrated the highest efficiency, exceeding 11% [6,7,8].

The methods used to synthesize organic or metal complex-based dyes involve complex procedures that include multiple steps and expensive chromatographic purification processes [9,10]. Switching from synthetic–organic and inorganic dyes to natural pigments like anthocyanin and chlorophyll, which can be readily derived from plant leaves, fruits, flowers and roots, could certainly address the aforementioned challenges [11,12]. Chlorophyll pigments are typically found in a wide variety of plant components, and each molecule of chlorophyll has four pyrrole rings surrounding a Mg^2+^ ion, among which one is attached to a phytol tail [13,14]. Chlorophylls are divided into two types, called chlorophyll a and b, that vary in one of the pyrrole rings’ at C3 positions in their structures. In chlorophyll b, the formyl (-CHO) chain is attached to the C3 side of the pyrrole ring, but in chlorophyll a, the methyl (-CH_3_) group is attached at the same location [15,16]. The different substituents that make up the chlorophyll a and b pigments result in different light absorbance characteristics [17,18]. Therefore, a broad range of wavelengths that correspond to the visible spectrum’s violet, red and blue portions are absorbed by chlorophyll and its derivatives [19,20]. Current state-of-the-art DSSCs are defined by solar-to-electric power conversion efficiencies (PCEs) of 10–13%, with a range of 350–750 nm. The primary barrier to these systems’ ability to boost their efficiency further is their lack of absorption in the far-red/near-IR region. The visible radiation (between 350 and 700 nm) makes up about 45% of solar energy that reaches the Earth’s surface, whereas red/NIR radiation (between 600 and 1000 nm) makes up around 25%. Thus, the higher current output of the device might be attained by the rational design of materials and molecules for maximum utilization of the solar spectrum [21,22].

Recently, a number of studies have reported that the photovoltaic performance of DSSCs could be rationally tuned by using different natural dyes [23,24,25]. Wongcharee and his group used natural dyes extracted from blue pea and rosella, as well as a combination of the aforementioned dye extracts, and created three distinct types of DSSCs. The light absorption spectrum of the dye mixture revealed peaks that matched with the distinct natural pigments found in blue pea and rosella. In contrast to the individual component dyes, there was no synergistic photosensitization effect or light absorption from the dye mixture adsorbed on TiO_2_ [26]. In a different study, Sengupta and his group extracted dyes from fresh spinach leaves and beetroots and used them to measure photovoltaic performance and achieved 0.29% efficiency with it. This was possible because of the absorbance characteristics of the extracted dyes over a wide range of solar energy [27]. Similarly, Park and his group extracted two separate color dyes (yellow and blue) from Gardenia Jasminoide Elli’s flowers. The mixture of both dyes shows an increase in the wavelength of light absorption than in specific dyes, which results in higher photovoltaic performance of the device [28]. Despite all these attempts, a lot of efforts are still underway across the globe by different researchers to identify new natural dyes in order to make them competitive in performance with synthetic dyes.

In the quest for natural sources for extracting efficient dyes, Rhodobacter Sphaeroides photobacteria (Rh. Sphaeroides) appear to be a very good choice. Rh. Sphaeroides belongs to the Rhodobacteraceae family and is commonly found in both fresh and marine water. Notably, the ability of Rh. Sphaeroides to perform photosynthesis by trapping sunlight has been already well established. It can absorb light and use a mechanism known as anoxygenic photosynthesis to transform the absorbed light into chemical energy because it has pigments called bacteriochlorophylls. Impressed with these characteristics, in this present work, we intended to extract Bacteriopheophytine-a (Bhcl) dye from Rh. Sphaeroides and to synthesize two cationic derivatives, viz. Guanidino-bacteriopheophorbide-a (Gua-Bhcl) and (2-aminoethyl)triphenylphosphono-bacteriopheophorbide-a (2AETPPh-Bhcl), and thereafter to fabricate DSSCs by employing all three compounds as natural sensitizers. The performance of the obtained DSSCs has been probed and correlated with the photophysical and electrochemical properties of the prepared dyes. 

## 2. Results and Discussion

### 2.1. Structural and Optical Characterization of Bhcl and Its Cationic Derivatives

**Figure 1:**
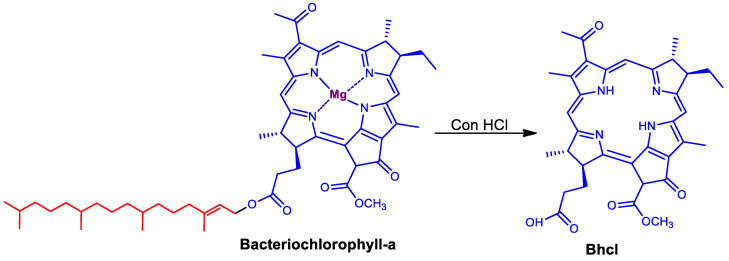
Isolation of bacteriopheophytine-a from Rh. Sphaeroides via Bacteriochlorophyll-a.

**Figure 2:**
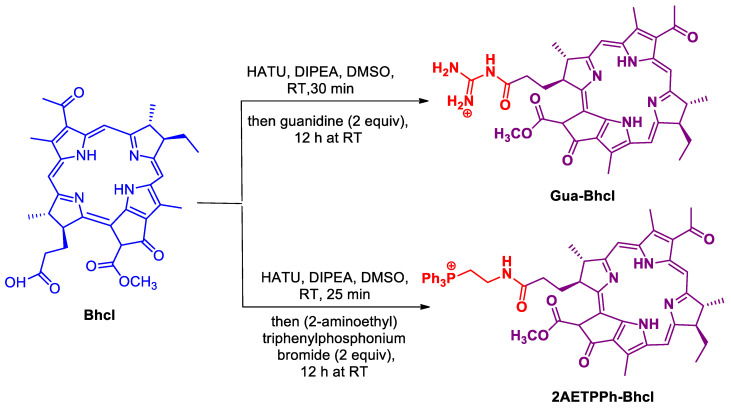
Synthesis of cationic derivatives of Gua-Bhcl and 2AETPPH-Bhcl.

The near-infrared natural pigment Bhcl was extracted from the bacteria Rh. Sphaeroides (Figure 1), and two cationic derivatives of this Bhcl were subsequently synthesized (Figure 2). The Gua-Bhcl contains positively charged mono-guanidinium moiety at the carboxyl terminal, whereas the 2AETPPh-Bhcl contains positively charged triphenyl phosphine moiety at the carboxyl terminal (Figure 2). It can be observed from Figure 2 that the carboxylic acid of Bhcl has been coupled with guanidine and (2-aminoethyl)triphenylphosphonium bromide in the presence of hexafluorophosphate azabenzotriazole tetramethyl uronium (HATU) as a coupling agent to obtain the mono-guanidinium and triphenyl phosphonium derivative of Bhcl, namely Gua-Bhcl and 2AETPPh-Bhcl, respectively. The LCMS analysis indicates that the isolated product with a molecular weight of 611.3 (M+1) corresponds to Bhcl (Figure S1). The product with a molecular weight of 653.7 (M+1) is the cationic derivate Gua-Bhcl (Figure S2), and that with a molecular weight of 899.06 (M-H_2_O) is the cationic derivative 2AETPPh-Bhcl (Figure S3).

**Figure 3:**
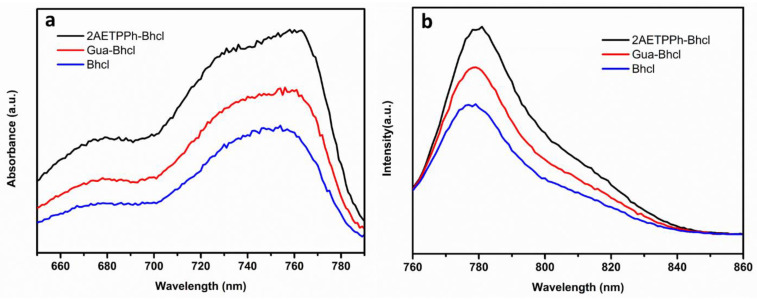
(**a**) Absorbance and (**b**) emission spectra of Bhcl, Gua-Bhcl and 2AETPPh-Bhcl.

Further, the photophysical properties of the extracted dye and its derivatives were investigated by recording their absorbance and emission spectra. The natural dye solution was initially prepared by dissolving 1 mM of dye (2AETPPh-Bhcl, Glu-Bhcl and Bhcl) in deionized water. Both UV-visible absorption and fluorescence emission studies were recorded at room temperature in a 1 cm × 1 cm quartz cuvette. As shown in Figure 3, when dissolved in methanol, at 1 μM concentration, Bhcl exhibits excitation and emission spectra around 775 and 796 nm, respectively. Further, the positively charged Bhcl derivative, Gua-Bhcl exhibits excitation and emission spectra around 756 and 779 nm, and the 2AETPPh-Bhcl exhibits excitation and emission spectra near 754 and 779 nm, respectively. These results infer that the extracted Bhcl and its cationic derivatives exhibit very strong excitation and emission in the NIR region and are thus regarded as potential candidates to be explored as sensitizers in DSSCs. Moreover, the positively charged guanidine and triphenylphosphine group in the derivatives might facilitate firm anchoring of the photosensitizer on the TiO_2_ surface to improve the solar energy conversion.

### 2.2. Photovoltaic Performance

**Figure 4:**
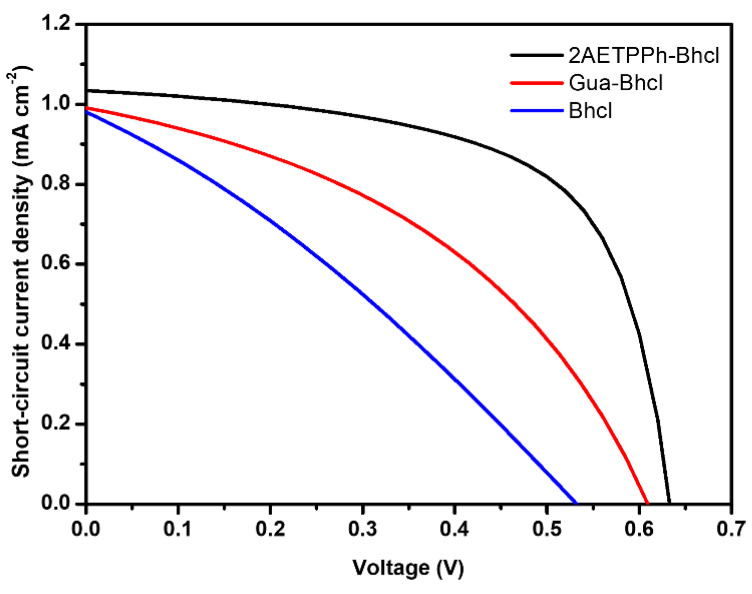
J–V curves obtained for DSSCs sensitized with Bhcl, Gua-Bhcl and 2AETPPh-Bhcl.

The interesting optical performance displayed by Bhcl and its derivatives motivated us to explore their ability as sensitizers in DSSC. Thus, the photovoltaic characteristics of the photoelectrodes fabricated using these new dyes were probed employing a Keithley 4200 source meter. The lamp intensity was kept at 100 mW cm^−2^ with an air mass 1.5 filter and the active area of the device was set to 0.25 cm^2^. Figure 4 depicts the typical current–voltage curve of the DSSC sensitized using Bhcl, Gua-Bhcl and 2AETPPh-Bhcl dyes. The photovoltaic parameters obtained for the constructed DSSCs are presented in Table 1, and it can be observed that all three dyes under investigation exhibit reasonable performance as sensitizers towards the construction of DSSCs. The Bhcl dye isolated from the bacteriochlorophyll displayed reasonably good short circuit photocurrent density (*J*_sc_), open circuit photovoltage (*V*_oc_), filling factor (FF) and efficiency (*η*) values of 0.97 mA cm^−2^, 0.52 V, 0.56 and 0.18%, respectively, obtained with the DSSC sensitized using Bhcl dye. Meanwhile, the derivative Gua-Bhcl exhibited *J*_sc_, *V*_oc_, FF and *η* values of 0.99 mA cm^−2^, 0.60 V, 0.60 and 0.25%, respectively, and the derivative 2AETPPh-Bhcl showed *J*_sc_, *V*_oc_, FF and *η* values of 1.03 mA cm^−2^, 0.63 V, 0.75 and 0.38%, respectively. Though all the compounds have shown their ability as sensitizers in DSSC, the Gua-Bhcl and 2AETPPh-Bhcl derivatives displayed higher performance than those of Bhcl. This could be because of the cationic charges available on these derivatives, which ensure a uniform dispersion and firm anchoring on the TiO_2_ photoanodes. Especially, the 2AETPPh-Bhcl derivative demonstrated excellent photovoltaic parameters compared to the Gua-Bhcl derivative, and this could be attributed to the presence of triphenylphosphine moiety, which can induce steric hindrance and, in turn, could efficiently enhance the open circuit photovoltage through minimization of the recombination effect. The photovoltaic performance investigations undoubtedly reveal that the isolated Bhcl and the cationic derivatives could be employed as sensitizers for the fabrication of highly efficient DSSCs. The maximum efficiency that could be attained is 0.38% with 2AETPPh-Bhcl derivative as the sensitizer, which is relatively less than many of the synthetic dye-based DSSCs. Even devices fabricated without the presence of any dye have been afforded an efficiency of 0.13% [25]. This efficiency could be significantly improved by the appropriate choice of photoanodes, counter electrodes and semisolid electrolytes, and this work is underway in our laboratory.

The stability of all three dyes was measured by performing current voltage (i–V) measurements for 10 days (Figure S4). During the three dyes, 2AETPPh-Bhcl displayed higher stability, which retains 29% of efficiency after 10 days compared to Gua-Bhcl (3.44%) and Bhcl (0.5%). The relatively better stability achieved with 2AETPPh-Bhcl could be due to the uniform and firm anchoring of the dye on the TiO_2_ photoanode.

### 2.3. Atomic Force Microscopic Studies

**Figure 5:**
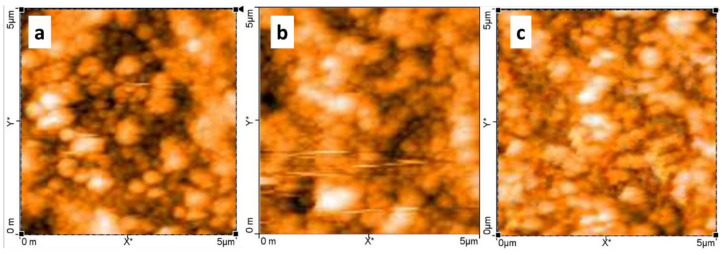
AFM images of TiO_2_ photoanodes sensitized using (**a**) Bhcl, (**b**) Gua-Bhcl and (**c**) 2AETPPh-Bhcl dyes.

The coverage of the dye molecules on the surface of the photoanode is a significant factor that decides the final output of the fabricated device. In order to investigate the dispersion of the dyes and their impact on the photovoltaic performance, we envisaged to probe the TiO_2_ photoanodes using atomic force microscopy (AFM). To develop TiO_2_-coated films, TiO_2_ was deposited on an FTO plate and calcined at 500 °C, after which the obtained film was immersed in the respective dye. Thereafter, the film was dried and then washed with water and ethanol and subjected to AFM measurements. The topography images of TiO_2_ sensitized with Bhcl, Gua-Bhcl and 2AETPPh-Bhcl dyes were recorded and presented in Figure 5. As depicted in Figure 5, a higher degree of inhomogeneity can be observed in the photoanode surface, which is sensitized with the Bhcl dye (Figure 5a). A relatively better surface coverage could be observed in the photoanode surface sensitized with Gua-Bhcl (Figure 5b). Notably, an ample surface coverage indicative of a uniform dispersion can be seen in the 2AETPPh-Bhcl dye-sensitized TiO_2_ photoanode (Figure 5c). The relatively better surface coverage observed with Gua-Bhcl and 2AETPPh-Bhcl dyes is again ascribed to the electrostatic interaction between the positive charge on these dyes and the photoanodes. The surface roughness factor of the dye-loaded TiO_2_ photoanode was calculated to be 78 nm, 95 nm and 115 nm for Bhcl, Gua-Bhcl and 2AETPPh-Bhcl, respectively. Thus, it can be concluded that the higher degree of coverage in the 2AETPPh-Bhcl dye-sensitized TiO_2_ has led to the enhanced performance of the constructed DSSC device than that observed with Bhcl and Gua-Bhcl. These results are well in agreement with the photovoltaic parameters recorded using J–V curves.

The photoconversion efficiency of the DSSC is dependent on several factors, which include the wettability of the dye, charge polarization of the dye and also the amount of dye loading on the photoanode. Higher loading of the dye on the photoanode can establish a greater number of electron injections into the conduction band of the TiO_2_, thereby delivering more carrier diffusion across the device [26]. As observed from our AFM images, the AETPPh-Bhcl dye displayed excellent and uniform loading on the TiO_2_ surface. The roughness factor of the dye-loaded TiO_2_ is also a key factor that decides the rate of photoconversion since the surface texture of the photoanode can influence the photon-reflecting angle and it can make the photon bounce back and deliver higher current generation indirectly. Here, the higher roughness factor (115 nm) of AETPPh-Bhcl-sensitized TiO_2_ delivers maximum photocurrent compared to the other two counterparts.

### 2.4. Electrochemical Investigations

**Figure 6:**
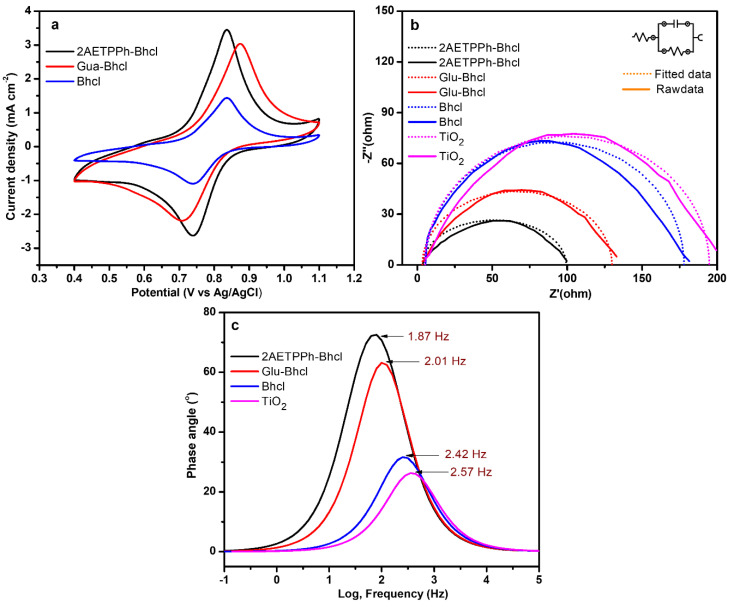
(**a**) Cyclic voltammograms of Ni foam with Bhcl, Gua-Bhcl and 2AETPPh-Bhcl (electrolyte: 2 M KOH). (**b**) Electrochemical impedance spectra and (**c**) Bode plots of DSSCs sensitized with Bhcl, Gua-Bhcl and 2AETPPh-Bhcl dyes.

In order to understand the performances of the Bhcl dye and its derivatives as sensitizers in DSSC, their electrochemical properties, such as redox behavior, and their conductivity were investigated by performing cyclic voltammetry (CV) and electrochemical impedance spectroscopy (EIS). The CV analysis of all three dyes was carried out employing nickel foam as the working electrode, Pt as the counter electrode, Ag/AgCl as the reference electrode and 2 M KOH as the electrolyte using a three-electrode setup with a PGSTAT204 Autolab workstation (Metrohom, The Netherlands). As observed from Figure 6a, all three dyes exhibit well-defined oxidation/reduction peaks indicative of their excellent redox behavior, and because of this, all dyes have shown reasonable photovoltaic performances. However, upon comparison, the Gua-Bhcl and 2AETPPh-Bhcl dyes display a better voltammetric response with higher current density when compared to that of Bhcl. The presence of cationic substituents in the Gua-Bhcl and 2AETPPh-Bhcl might have facilitated electron shuttling, which resulted in higher current densities. This, in turn, is reflected in the higher photovoltaic performance of Gua-Bhcl and 2AETPPh-Bhcl than that observed with Bhcl.

EIS was employed mainly to probe the interfacial kinetics and charge transport properties of the fabricated DSSCs. The EIS plot of DSSC contains three main regions, in which the higher frequency region denotes the resistance between the FTO and TiO_2_ interface [29,30]. The lower frequency region denotes the charge transfer resistance between the counter electrode and electrolyte interface and the middle portion denotes the recombination resistance between the dye and electrolyte. A smaller semicircle represents the low recombination as well as higher charge kinetics, and this would facilitate the performance of DSSC [31,32]. The EIS spectra of the unmodified TiO_2_ and the dye-coated TiO_2_ have been recorded (Figure 6b). It could be observed that the unmodified TiO_2_ exhibits a very high *R*_ct_ value of 190 Ω, which could be due to the high repulsion between the TiO_2_ photoanode and the negatively charged iodide ions in the electrolyte. Upon coating with Bhcl, the *R*_ct_ value (173 Ω) did not change significantly. However, upon coating with Gua-Bhcl and 2AETPPh-Bhcl, the *R*_ct_ value reduced drastically to 126 Ω and 96 Ω, respectively. Since the positively charged dyes Gua-Bhcl and 2AETPPh-Bhcl anchor on the TiO_2_ photoanodes, the repulsion between the electrolyte and photoanode might have significantly reduced, thus resulting in a reduced *R*_ct_ value. Likewise, the solution resistance (*R*_s_) values of 2AETPPh-Bhcl, Gua-Bhcl, Bhcl and unmodified TiO_2_-based devices were found to be 3.21, 3.56, 5.5 and 5.05 Ω, respectively. The low *R*_s_ and *R*_ct_ value of a 2AETPPh-Bhcl-based device denotes the low recombination and high conductivity, which lead to enhanced photovoltaic performance.

From the Bode plot, it can be seen that the Gua-Bhcl and 2AETPPh-Bhcl dye-sensitized DSSC devices showcase a lower frequency shift along with a greater phase angle, which depicts the longer electron lifetime of the constructed device compared to the Bhcl and bare TiO_2_-based DSSC [33,34]. The electron lifetime obtained using the equation τ_e_ = (2πf_max_)^−1^ for TiO_2_, Bhcl, Gua-Bhcl and 2AETPPh-Bhcl dyes were found to be 61.95 ms, 66.20 ms, 79.17 ms and 85.05 ms, respectively. The maximum electron lifetime observed with 2AETPPh-Bhcl dye-based DSSC (compared to Bhcl and Gua-Bhcl) suggests the lower recombination rates due to the bulkier triphenylphosphine moiety, thereby enhancing the electron density and photovoltaic performance of the fabricated DSSC.

**Figure 7:**
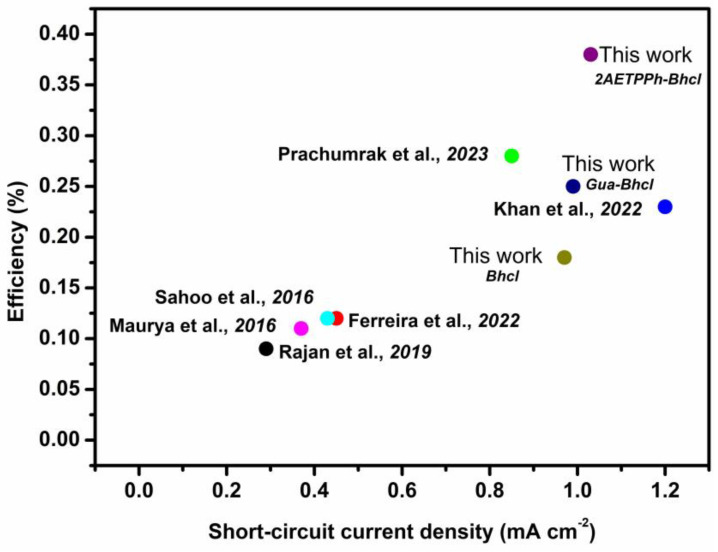
Comparative photovoltaic performance of DSSCs sensitized using natural dyes [35,36,37,38,39,40].

Overall, the photovoltaic performance of DSSCs sensitized with Bhcl, Gua-Bhcl and 2AETPPh-Bhcl are comparable to or better than many of the recently reported natural dyes, and the performances are compared in Figure 7.

## 3. Materials and Methods

### 3.1. Chemicals

2-(1H-7-Azabenzotriazol-1-yl)-1,1,3,3-tetramethyl uronium hexafluorophosphate methanaminium (HATU) was obtained from Genscript Inc. (Piscataway, NJ, USA). Guanidine hydrochloride, (2-aminoethyl) triphenylphosphonium bromide, diisopropylethylamine (DIPEA), dimethyl sulfoxide (DMSO) and all other reagents were purchased from Sigma-Aldrich. 

### 3.2. Characterization

The extracted dye and their derivatives were characterized using triple quadrupole mass spectrometer (Shimadzu LCMS-8060: Kyoto, Japan). Agilent Cary 60 UV–Vis spectrophotometer was used to record the absorption spectra of the dye. For the emission spectra, Agilent Cary Eclipse Fluorescence Spectrophotometer with an inbuilt pulsed diode LASER was used for excitation. Atomic force microscopy (AFM-Park XE7, in 1 µm × 1 µm scan area, Yongin City, South Korea) was used to analyze the dye loading and uniformity of the photoanode surface. Compounds were purified by preparative reverse phase (RP)-HPLC (Waters, xTerra C18 10 μm; 19 mm × 250 mm, Granada, Spain) and analyzed using UPLC (Acquity, BEH C18, 1.7 μm, 2.1 mm × 50 mm). Purity of the isolated compounds was analyzed by analytical HPLC using either achiral column (XSelect CHC PFP C18, 2.5 μm, 4.6 mm × 150 mm, USA) and chiral column [Chiralpak ZWIX(+) 3 μm, 3.0 mm × 150 mm] with solvent gradient of 3% B to 30% B in 30 min with run time of 45 min (where A = 30 mM ammonium acetate and B = acetonitrile) unless otherwise noted. All the isolated compounds demonstrated ≥95% purity at λ = 270 and/or 770 nm in the aforementioned analytical HPLC method.

### 3.3. Isolation of Bacteriopheophytine-a (Bhcl) from Rh. Sphaeroides

In the typical extraction process, Rh. Sphaeroides was added to 1-propanol with continuous N_2_ flow under stirring condition for 12 h at room temperature. The obtained mixture was filtered under vacuum using Whatman no. 1 filter paper, and then the residue was washed several times with 1-propanol until the solution became colorless. The obtained extract was labeled as bacteriochlorophyll-a and the product was confirmed by LCMS analysis with a molecular weight of 911.1 (M + 1). In order to obtain bacteriopheophytine-a (Bhcl), 20 mL of con. HCl was added to the colorless solution and stirred for another 20 min (Figure 1). Afterwards, the solution was transferred to 5% NaCl (1.5 L) and purified with CH_2_Cl_2_ using silica column, to obtain the Bhcl product. The obtained Bhcl was confirmed by LCMS analysis (Figure S1) and the isolated product was obtained with a molecular weight of 611.3 (M + 1).

### 3.4. Synthesis of Cationic Derivatives of Bhcl

For the synthesis of the cationic derivatives, Bhcl, diisopropylethylamine and HATU were taken in 1:5:1 proportion in dimethyl sulfoxide and the mixture was subjected to stirring under Ar flow for 30 min. To synthesize Guanidino-bacteriopheophorbide-a, thrice the amount of guanidine hydrochloride salt was added to the reaction mixture and the stirring continued at RT overnight. Similarly, in order to obtain (2-aminoethyl)triphenylphosphono-bacteriopheophorbide-a, thrice the amount of (2-aminoethyl)triphenylphosphonium bromide was added to the reaction mixture and the stirring continued overnight at room temperature. Thereafter, the obtained products were subjected to purification using preparative reverse-phase HPLC comprising a mobile phase of A = 20 mM ammonium acetate buffer at pH 7 and B = acetonitrile; gradient 0–50% B in 30 min, 13 mLmin^−1^, λ = 280 nm. Finally, the purified fractions were analyzed using LCMS (Figures S2 and S3), then pooled and lyophilized to furnish Gua-Bhcl [653.7 (M + 1)] and 2AETPPh-Bhcl [899.06 (M-H_2_O)].

### 3.5. Electrochemical Techniques 

The electrochemical investigations including cyclic voltammetry (CV) and electrochemical impedance spectroscopy (EIS) were performed with three-electrode setup with PGSTAT204 Autolab workstation (Metrohom, The Netherlands). CV was carried out employing nickel foam as working electrode, Pt as counter, Ag/AgCl as reference electrodes and 2 M KOH as the electrolyte. The frequency range for the EIS was set as 10^−2^ to 10^5^ Hz. A bias voltage of 0 V is applied in dark condition to the fabricated DSSC devices.

### 3.6. Device Fabrication

Initially, glass substrates covered with fluorine-doped tin oxide (FTO) (surface resistivity = 7.5 Ω cm^−2^) were cleaned by sonication in acetone and ethanol for around 15 min each. To make a semisolid TiO_2_ slurry, 100 mg of P25-TiO_2_ was ground using an agate mortar and pestle with a few drops of water, acetylacetone and Triton X-100. To construct TiO_2_-coated thin films, TiO_2_ slurry was coated over FTO plate using doctor blading method followed by calcination for 30 min at 500 °C. Further, the constructed TiO_2_ thin films were immersed separately in the respective extracted natural dye. The dye-coated thin films were then dried and washed with water and ethanol. The photoanode was coated with the respective dye (with thickness of 12 µm) and the counter electrode on another FTO substrate was coated with platinum (Pt) (with thickness of 5 nm). The counter electrode was prepared as follows: 0.01 M of H_2_PtCl_6_ was dissolved in isopropanol and the obtained homogeneous solution was drop-casted on a precleaned FTO, which was annealed at 450 °C for 30 min to obtain Pt CE. Subsequently, the DSSC device was generated upon injection of a minute quantity of I^−^/I_3_^−^ electrolyte in between the photocathode and the dye-coated photoanode.

## 4. Conclusions

In summary, the Bacteriopheophytine-a (Bhcl) dye was extracted from Rh. Sphaeroides photobacteria and two of its derivatives, viz. Gua-Bhcl and 2AETPPh-Bhcl, which were synthesized and employed as natural sensitizers for the construction of efficient DSSCs. The photophysical investigation of Bhcl and the cationic derivatives Gua-Bhcl and 2AETPPh-Bhcl showed excellent absorbance around the NIR region. The photovoltaic performance of all three dyes was probed, among which the DSSCs sensitized with the cationic derivatives Gua-Bhcl and 2AETPPh-Bhcl exhibited better performance, which could be due to the uniform dispersion of the cationic dyes on the TiO_2_ photoanodes through electrostatic interaction. Especially, the 2AETPPh-Bhcl demonstrated superior photovoltaic performance with an efficiency of 0.38% at *J*_SC_ of 1.03 mA cm^−2^, and this performance is comparable to or better than many of the naturally obtained dyes. The topological investigations using AFM and electrochemical studies using CV and EIS also evidence the superior performance obtained with 2AETPPh-Bhcl dye as the sensitizer. This investigation paves the way for the extraction of more natural dyes and designing task-specific derivatives for the construction of DSSCs and other optical devices.

## Figures and Tables

**Table 1:** Photovoltaic parameters of fabricated DSSCs sensitized using different dyes.

Parameters	Bhcl	Gua-Bhcl	2AETPPh-Bhcl
*V*_oc_ (V)	0.52	0.60	0.63
*J*_sc_ (mA cm^−2^)	0.97	0.99	1.03
FF	0.56	0.60	0.75
*η* (%)	0.18	0.25	0.38

## Data Availability

Data are contained within the article and Supplementary Materials.

## Supplementary Materials

The following information is given in the supporting information at: https://www.mdpi.com/article/10.3390/molecules29050931/s1. Figure S1: LCMS of Bhcl; Figure S2: LCMS of Gua-Bhcl; Figure S3: LCMS of 2AETPPh-Bhcl; Figure S4. Stability of the DSSCs sensitized with different dyes. Table S1: Comparative photovoltaic performance of DSSCs sensitized using natural dyes.
