# Supplementary material for: Highly Efficient DSSCs Sensitized Using NIR Responsive Bacteriopheophytine-a and Its Derivatives Extracted from Rhodobacter Sphaeroides Photobacteria
Source: Molecules. 2024 Feb 21;29(5):931. doi: 10.3390/molecules29050931 (PMC10935420; doi:10.3390/molecules29050931)
Supplement: Supplementary file 1 [file molecules-29-00931-s001.zip › molecules-2826790-supplementary.pdf]

Supplementary Material

Highly Efficient DSSCs Sensitized using NIR Responsive Bacteriopheophytine-a and its Derivatives Extracted from Rhodobacter Sphaeroides Photobacteria

Abdulrahman I. Almansour, Raju Suresh Kumar,* Khloud Ibrahim Al-Shemaimari and Natarajan Arumugam*

Department of Chemistry, College of Science, King Saud University, P.O. Box 2455, Riyadh 11451, Saudi Arabia.

**Correspondence: sraju@ksu.edu.sa (Raju Suresh Kumar); anatarajan@ksu.edu.sa (Natarajan Arumugam)

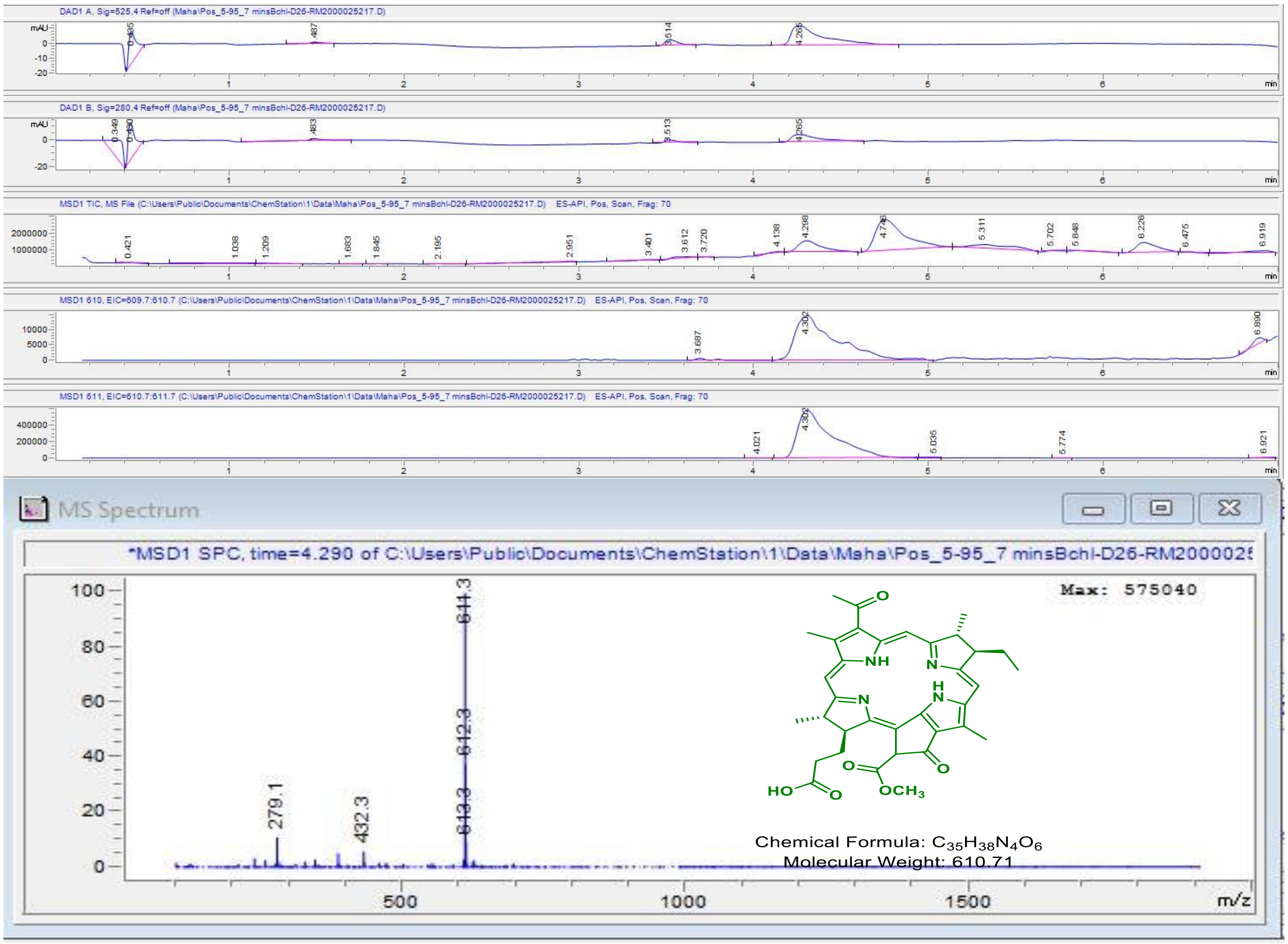

Figure S1. LCMS of bacteriochlorophyll-a (Bchl).

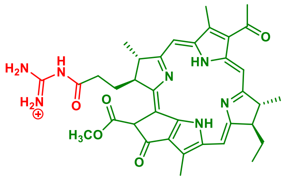

Gua-Bhcl (3)

Chemical Formula: $C_{36}H_{42}N_7O_5^+$

Molecular Weight: 652.78

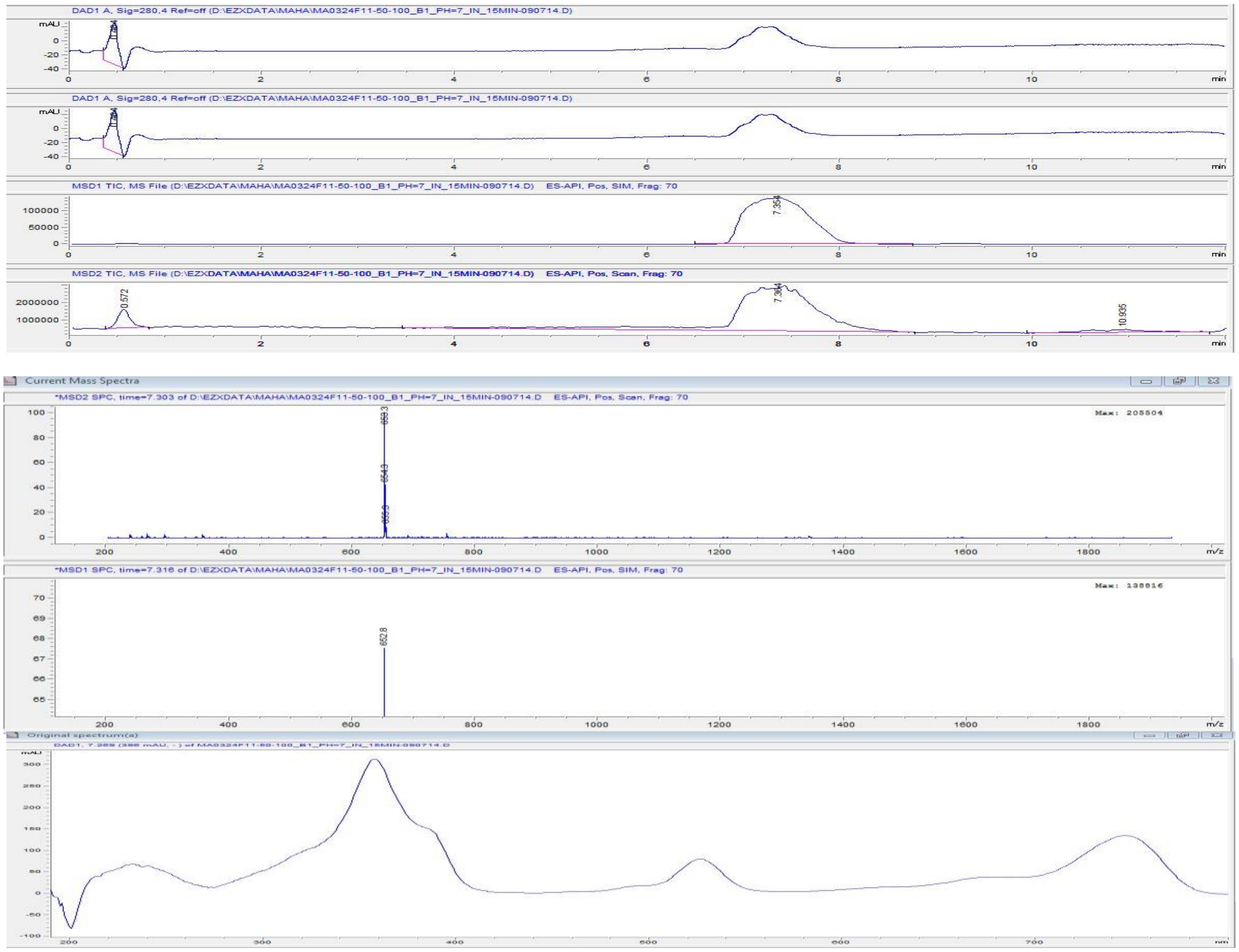

Figure S2. LCMS of Gua-Bhcl.

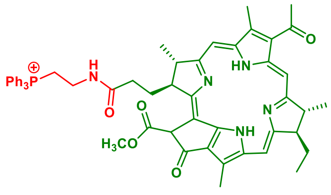

TPP-Bhcl (4)

Chemical Formula: $C_{55}H_{57}N_5O_5P^+$

Molecular Weight: 899.06

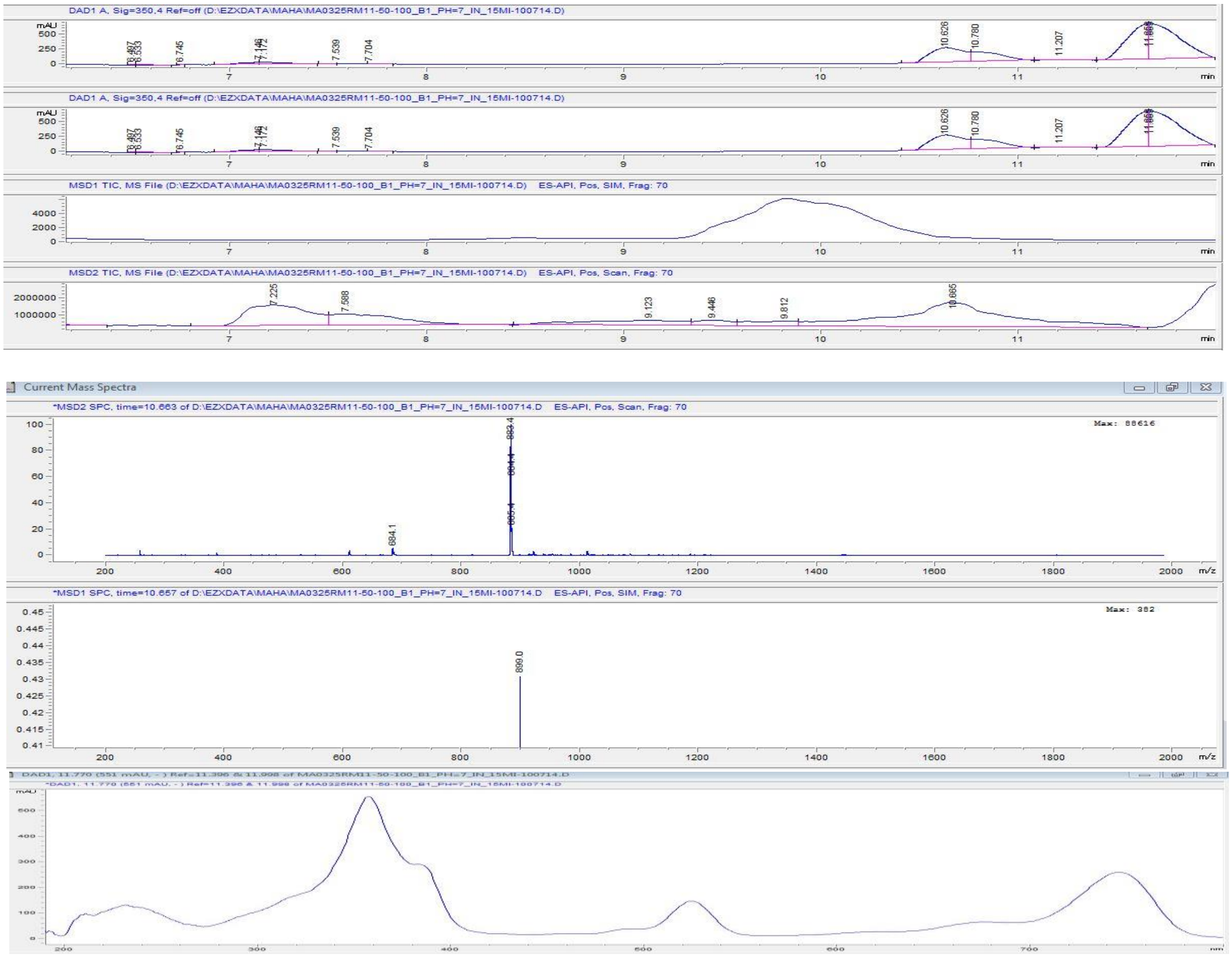

Figure S3. LCMS of 2AETPPH-Bhcl.

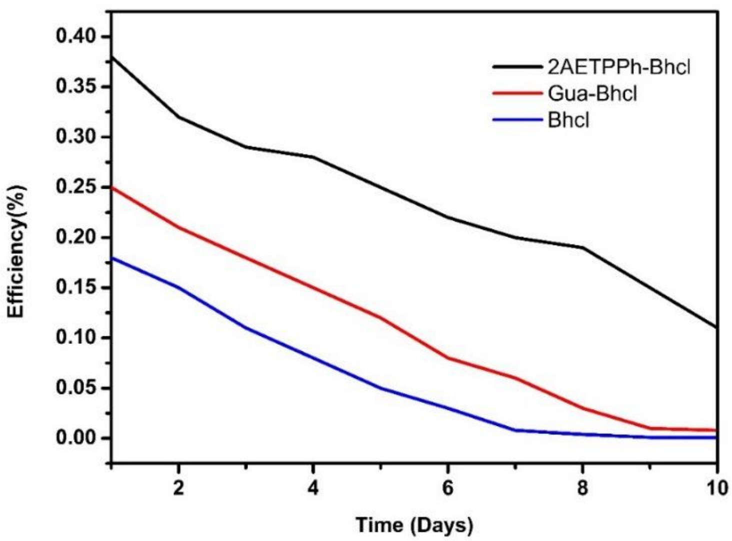

Figure S4. Stability of the DSSCs sensitized with different dyes.

Table S1 Comparative photovoltaic performance of DSSCs sensitized using natural dyes

Source	J_{SC} (mA cm^{-2})	V_{OC} (V)	FF	PCE (%)	Ref
Saraca asoca (flowers)	0.29	0.51	0.51	0.09	35
Lantana repens (flowers)	0.45	0.69	0.34	0.12	36
Nymphaea pubescens Willd. (flowers)	0.85	0.52	0.63	0.28	37
Mitragyna speciose (leaves)	1.20	0.52	36.7	0.23	38
Canna lily yellow (flowers)	0.43	0.56	0.40	0.12	39
Indigofera tinctorial (leaves)	0.37	0.48	0.63	0.11	40
Strobilanthes cusia (leaves)	0.003	0.28	0.25	0.01	41
Bhcl (from sea algae)	0.97	0.52	0.56	0.18	This work
Gua-Bhcl (derived from Bhcl)	0.99	0.60	0.60	0.25	This work
2AETPPh-Bhcl (derived from Bhcl)	1.03	0.63	0.75	0.38	This work
